# The Influence of Educational Determinants on Children’s Health: A Scoping Review of Reviews

**DOI:** 10.3389/phrs.2024.1606372

**Published:** 2024-06-05

**Authors:** Amandine Fillol, Louise Wallerich, Marie-Pier Larose, Christine Ferron, Ana Rivadeneyra-Sicilia, Stéphanie Vandentorren, Jessica Brandler-Weinreb, Linda Cambon

**Affiliations:** ^1^ University of Bordeaux, Institut National de la Santé et de la Recherche Médicale (NSERM), Bordeaux Population Health (BPH), U1219, Méthodes de Recherche Interventionnelle pour la Santé des Populations (MéRISP), Population Health Translational Research (PHARES)/Equipe Labellisée Ligue Contre le Cancer, Centre d’Investigation Clinique 1401/Centre Hospitalier Universitaire de Bordeaux, Service de Prévention, Institut de Santé Publique, d’Épidémiologie et de Développement (ISPED)/Chaire Prévention, Bordeaux, France; ^2^ INVEST Flagship Research Center, Department of Psychology and Speech-Language Pathology, University of Turku, Turku, Finland; ^3^ Fédération Promotion Santé, Auberviliers, France; ^4^ University of Bordeaux, INSERM, BPH, U1219/PHARES, Bordeaux, France; ^5^ Santé Publique France, Paris, France

**Keywords:** children, social determinansts of health, education, scoping review, health in all policies

## Abstract

**Objectives:** Education is one of the most important social determinants shaping the development and wellbeing of children. The purpose of this review of reviews is to inform policymakers, practitioners and public health stakeholder involved in developing child-friendly policies outside of the healthcare system.

**Methods:** We carried out a scoping review of reviews. It included 32 reviews.

**Results:** We identified four main categories of educational determinants in relation to children’s health: 1) the organization and structure of educational activities, 2) the interpersonal relations in the educational facilities and structures, 3) the spatial environment of educational facilities and structures, 4) social inequalities in the educational facilities and structures. This last category highlighted the capacity of education system to act on inequalities derived from the way social structures are organized.

**Conclusion:** We suggest a conceptual framework for action which distinguishes structural determinant (gender, race, social class, etc.) and structuring determinant (public policy, systems of governance, organization of cultures/values consideration). Finally, we discuss on how these social structures and structuring determinants influence the intermediary educational determinants collated in the review.

## Introduction

The first few years of life have a crucial impact on the health of children, and their future wellbeing as adults. Investment in children’s health is not only a matter of social justice [1], it is also sound economic and societal strategy since investing in children actually delivers economic benefits throughout the rest of their life cycle (health expenditure, employability, productivity, etc.) [2].

Education, in particular, is one of the most important social determinants shaping the development and wellbeing of children [3]. Education can be defined as a process, formal or informal, which intentionally targets children’s capacities to absorb knowledge, skills and values, and to manage their emotions and social relations. These capacities enable children to become autonomous, engaged, accomplished members of society [4]. Education has a direct impact on health indicators such as life expectancy, as well as certain health-related behaviors (e.g., smoking, diet, sexual health) and medical problems (e.g., depression, obesity, chronic illnesses) [5]. For example, high-quality early childhood education and care (ECEC) settings (for children aged 0–6) can help children to improve their self-regulation skills, their capacity to form relationships, knowledge acquisition and the development of specific social, motor and cognitive skills [6], with long-term consequences for their physical health and life skills [7, 8]. Through its holistic approach to child development, high-quality ECEC can promote child school readiness, and is reported particularly beneficial from children from low-income households [9–11]. It is important that educational facilities and structures are staffed with competent professionals, capable of fostering affectionate and reassuring interactions in surroundings which are safe, hygienic and accessible to parents. Groups size should allow good interaction among children and between children and adults, with effective and caring supervision to ensure educational cohesion [12].

There is no clear consensus regarding the form of educational interventions and policies required to create conditions conducive to good health in children. Despite the growing corpus of public health research aimed at developing models and frameworks to improve and strengthen the positive links between education and health, these studies are often largely overlooked or misunderstood by public policymakers. The purpose of this review is thus to provide an overview of the current state of knowledge regarding the determinants of education and their consequences on child health. This overview aims to inform policymakers, practitioners and public health stakeholders involved in developing child-friendly policies outside the healthcare system. In other words, the objective of this review is to identify the documented determinants within educational facilities and structures that is associated with the amelioration of children’s health.

## Methods

### Study Design

A scoping review of reviews can help providing a broad overview of the existing corpus of research into a topic. We adopted the methodology proposed by Arksey and O’Malley [13]. We used the Preferred Reporting Items for Systematic Reviews and Meta-analyses guidelines (PRISMA 2020) [14].

### Identifying the Research Question

This scoping review of reviews aimed at identifying the way educational determinants can improve children’s physical, cognitive, mental, and social health. To specify and focus our research questions, we used the PICoS (population-phenomena of interest-context-study design) framework, an adaptation of the PICO (population-intervention-comparison-outcomes) framework for reviews including qualitative reviews [15, 16].

#### P—Population

This review focuses on children from birth up to the end of elementary school (12 years old), without any diagnosed health conditions. There are two main categories of educational establishments associated with this age range: ECEC settings (ages 0–6) and primary schools (ages 6–12).

#### I—Phenomena of Interest

Our phenomena of interest are educational determinants and child health. Education can be defined either as a context or a process. It is associated with institutions which provide explicit and deliberate learning and activities which are aimed to develop social, physical, emotional or cognitive development. Education is also present in parent-child interactions, but we chose not to include these aspects since this review is part of a broader project which includes a review focusing specifically on parenting determinants. We adopted the definition of health proffered by the World Health Organization (WHO): “a state of complete physical, mental and social wellbeing and not merely the absence of disease or infirmity” [17].

#### C—Context

We chose to focus on structural educational determinants, rather than individual and biological determinants. By structural determinant, we mean the various systems that generate health inequities. Structural determinants are defined as “those that generate stratification and social class divisions in the society and that define individual socioeconomic position within hierarchies of power, prestige and access to resources.” [8] They include processes of governance, economic and social policies, cultural context and social structures that affect income, working conditions, housing, and education [18].

#### S—Study Design

We included all articles using a review method. As systematic methods are not used in all academic disciplines, we chose to also include all studies described as reviews by the authors or by the electronic databases.

#### Research Paradigm and Research Question

Our research has been thought in the pragmatic paradigm. This paradigm aims at utilizing the best methods to investigate real‐world problems and to provide an action‐oriented framework for research [19, 20]. This review is the first part of a scientific program which support decision-making by making an analytical approach to public policy on children’s health easier. Thus, our main research question is: what are the determinants providing good health in education facilities and structures?

### Identifying Relevant Studies

The search strategy was developed by the two first authors (AF, LW) with regular meetings with the other authors (M-PL, CD, LC). The final strategy was approved by a specialized librarian at the School of Public Health of the blind for review. The search was performed from July to September 2022. Search strategy included terms related to:

i) Institutions and activities explicitly concerned with learning (e.g., schools and child care facilities) and development (e.g., physical activities, music, creative activities).
ii) children’s health and development.
iii) review articles.

To capture as many relevant publications as possible, the list of terms was iteratively revised after searching the databases. The strategy was thus narrowed down by date of publication (between 2010 and 2022), language (French or English) and type of publication (peer-review) (Supplementary Additional File S1).

We searched the following electronic databases (Supplementary Additional File S2): PROQUEST (ERIC, International Bibliography of the Social Sciences (IBSS), Linguistics and Language Behavior Abstracts (LLBA), Political Science Database, Sociological abstract), EBSCOHOST (APA PsycInfo, APA PsycArticles, Psychology and Behavioral Sciences Collection, SocINDEX with Full Text, CINAHL Complete), Web of sciences, Pubmed. Reference lists of key publications were also manually searched by the review team. Covidence (a review software program) was then used to identify and screen the studies. The search also encompassed grey literature: OECD, the British Education Index, the Center for the Developing Child (Harvard), UNESCO, EURYCIDE, WHO.

### Study Selection

The study selection process consisted of three stages: 1) title screening, 2) title and abstract screening, 3) full-text screening. The full-text screening was performed by two reviewers (AF and LW). The inclusion and exclusion criteria are detailed in the table below (Table 1).

**TABLE 1:** Inclusion and exclusion criteria (APPIE study, Bordeaux, France, 2024).

Inclusion criteria
Population
Study is about children between 0 and 12 years old, or elementary school aged children
And Intervention/determinants
Study is about the quality of education systems
Or Study is about parental implication in educational setting or activity
Or Study is about inclusion of health topics in school programs
And Outcomes
Study outcomes are about children’s health
Or Study is about inclusion of children with specific needs in educational settings or activities from an organizational point of view (the capacity of setting to integrate specific needs)
And Design
Systematic methods to review the literature have been applied
Or Methods are described

Exclusion criteria
Population
Study is about children with specific needs
Or Intervention/determinants
Study is about family based educational child care
Or Study is about parental education
Or Study deals with therapeutic education or medical prevention
Or Study is about child welfare
Or Outcomes
Study outcomes are about illness
Or Study is about children with a severe illness or children with special needs
Or Study is about biological characteristics of child development
Or Study is about academic performance
Or Study is about methods used to analyze children’s health or educational determinants
Or Design
The article is a study protocol
Or The article is a meta-analysis

### Charting the Data

Synthesis and interpretation of data were performed by two reviewers (AF and LW) using a data extraction tool (Supplementary Additional File S3).

The first author (AF) utilized a three-stage process to analyze the results: 1) description of the reviews included in the sample, 2) thematic analysis of the reviews with reference to outcomes, research question and the main purpose of the scoping review of reviews. For the second step, an inductive analysis was used.

### Collating, Summarizing and Reporting the Results

We include an introductory section aiming at describing the reviews included in our selection. It includes a flowchart, as recommended in the PRISMA guidelines. In a second sub-section, we tackle our research question (association between determinants within educational facilities and structures and children’s health).

## Results

### Description of the Selected Sample of Reviews

**FIGURE 1:**
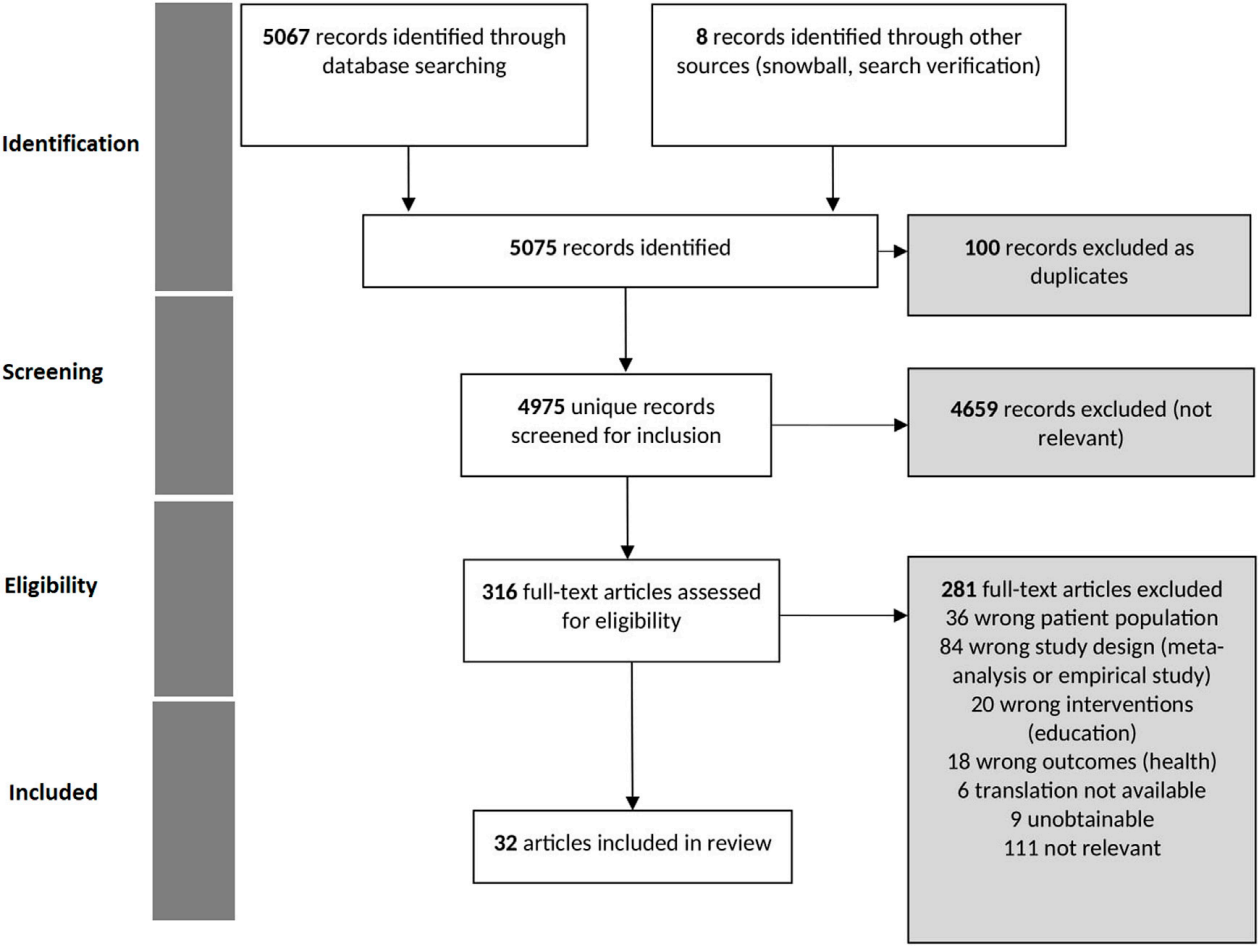
PRISMA flowchart of study selection process (APPIE study, Bordeaux, France, 2024).

The original search conducted from June to September 2022 yielded 5,067 potentially relevant articles. After duplication and relevance screening, 316 citations met the eligibility criteria based on title and abstract. The corresponding full-text articles were obtained for review: 36 were related to a patient population outside the scope of the review, 84 had a study design which did not meet the reviewing method criteria, 20 were centered on interventions which were not in the education sector, 18 had outcomes referring to another definition of health incompatible with the own from the WHO, 6 were not translated into French or English, 9 were unobtainable and 111 were not relevant. Thirty-two (32) articles were thus included in the final review (Figure 1).

We divided educational facilities and structures into several categories based on the study setting (Table 2), and their denomination in French and English. The full description of the articles is presented in Supplementary Additional File S4.

**TABLE 2:** Different types of educational structures (APPIE study, Bordeaux, France, 2024).

Age group	Educational structures		
	French speaking designation	English speaking designation	
0–3	Lieu d’accueil des jeunes enfants, crèches/Lieu d’accueil des jeunes enfants	Daycare	Early Childhood Education and Care (ECEC) setting
3–4	Ecole maternelle (petite section)/Lieu d’accueil des jeunes enfants	Preschool/Nursery	
4–5	Ecole maternelle (moyenne section)*/*Lieu d’accueil des jeunes enfants	Preschool/Prekindergarten	
5–6	Ecole maternelle (grande section)	Kindergarten	
6–12	Ecole primaire/élémentaire	Primary/Elementary school	

Almost half of the reviews identified were conducted between 2020 and 2022 (*n* = 16/32), with the clear majority published by lead authors attached to an American or European institution (*n* = 25/32). Half of the reviews focused on ECEC settings (ages 0–6) (*n* = 14/32). The majority of the reviews identified focused on the organization of educational activities (*n* = 15/32), with the least-discussed subject being the spatial environment of educational facilities and structures (*n* = 7/32) (Table 3).

**TABLE 3:** Properties of the reviews included in our scoping review (APPIE study, Bordeaux, France, 2024).

Properties	Number of reviews
Year	
2010–2015	7
2015–2020	9
2020–2022	16
Country of origin of lead author (institution)	
North America	13
Europe	12
Asia	2
Oceania	5
Setting	
Early childhood education and care settings	14
Primary schools	5
Early childhood education and care settings and primary schools (and/or secondary schools)	12
Not specified if the review is about early childhood education and care settings or primary school	1
Determinants of educational quality	
Organization and structure of educational activities	15
Interpersonal relations in the educational facilities and structures	6
Spatial environment of educational facilities and structures	4
Social inequalities in educational facilities and structures	7

### Association Between Educational Facilities and Structures and Children’s Health

Thematic analysis of these reviews revealed four major subjects of interest: 1) Organization and structuring of educational activities; 2) the interpersonal relations in the educational facilities and structures; 3) Spatial environment of educational facilities and structures; 4) Social inequalities in educational facilities and structures.

#### Organization and Structure of Educational Activities

##### The Teaching Program or Curriculum

Different educational innovations are described in the reviews:

• Social-emotional learning interventions: the acquisition of cognitive and non-cognitive skills, sometimes referred to as “socio-emotional skills” or “general skills” conducive to healthy development (interpersonal skills, responsible decision-making, self-awareness, social awareness and self-control) [21–24]);
• Mindfulness interventions: the capacity to “*intentional self-regulation of attention from moment to moment, and other definitions have since emerged centering on focal awareness of experience in the present moment*” (28: 1221) [25–27];
• Innovative educational formats such as learning-through-play and outdoor learning, the use of stories or brief interludes of physical activity during traditional sessions [21, 28–31].

In both ECEC settings and primary schools, the *social-emotional learning interventions* tend to focus on the regulation of emotions, problem-solving and coping skills, empathy, self-esteem, wellbeing, positive emotion, social capacities, building better relationships between teachers and pupils, satisfaction with one’s life and overall fulfilment, while helping to protect against depression, anxiety, stress, emotional problems and negative emotions, particularly among children exhibiting symptoms of psychological disorders [22, 23]. Studies analyzing *mindfulness interventions* have shown that such program can have beneficial effects for children, particularly by helping them to overcome emotional problems and negative emotions, and by boosting social and emotional skills, wellbeing and self-esteem, particularly in children with emotional problems or stress issues [22, 25]. *Yoga*, regarded as encompassing aspects of mindfulness and meditation as well as physical postures, breathing and relaxation techniques, may also have positive effects in terms of reducing anxiety, depression and inhibition, while encouraging feelings of physical and psychological wellbeing, self-esteem, resilience, attention and even educational performance [26]. Broadly speaking, such interventions appear to have a greater impact on emotional and behavioral problems and hyperactivity between the end of pre-school and the onset of adolescence [25]. For both social emotional learning interventions and mindfulness interventions, the reviews suggest that there is no clear consensus on the impact of these interventions on child health and that the existing research is in need of consolidation [24, 25, 27].

In primary schools, *innovative teaching methods* such as outdoor learning and learning through play, integrated with more traditional lessons, may boost children’s social involvement [22]. Outdoor learning may take many forms: outdoor adventure play, the creation of school gardens, excursions or the teaching of traditional subjects in natural surroundings [28]. Review conclude that these forms of learning can increase the level of engagement shown by pupils, conducive to better appropriation of lessons, stronger exam results and improved social and emotional skills, collaboration and self-esteem [21, 28] and dietary health [30]. However, the authors highlight the lack of established evidence for the connection between such activities and their expected health benefits. Innovative teaching methods like the use of stories by teachers can improve pupils’ language skills and create a friendly, respectful environment. Storytelling can also be a means of conveying messages about healthy behavior, lifestyle habits, dependency and psychosomatic troubles [31]. Innovative teaching methods like brief interludes of physical activity during traditional sessions can have a positive impact on the self-efficacy of children, improving the children’s physical conditions, fundamental movement skills, their quality of life and self-confidence, as well as the pleasure they take in physical activity [32–35].

##### Extra-Curricular Activities

Including physical activity among extra-curricular activities has been shown to have a positive impact on children’s depression, anxiety, stress and psychological distress. Participation in a variety of activities such as sport, dance and martial arts can boost self-efficacy, self-esteem, wellbeing and mental health, while reducing emotional problems, anxiety, negative emotions and symptoms of depression [22]. Team sport activities appear to have greater beneficial effects than individual sports. Other forms of organized activity such as youth organizations and arts groups have a positive impact on self-esteem, self-confidence, satisfaction in life and optimism [22]. Studies focusing on the practice of yoga and artistic activities outside of teaching hours have yielded contradictory results [25–27]. Interventions involving the creation of after-school clubs have been shown to improve social and emotional skills, with lasting effects when monitored over a period of 12 months [22].

Furthermore, informal learning activities may also be developed outside of school hours. For example, one review focusing on informal learning of STEM subjects (sciences, technology, engineering and mathematics) looked at the use of STEM skills to resolve everyday social problems, making for a more concrete and contextualized learning experience [36]. The review suggests that this learning approach shows great promise, particularly within family or community contexts, but there is still a lack of research into the potential effects of this form of learning on the socio-emotional capacities of pre-school children.

#### The Interpersonal Relations in the Educational Facilities and Structures

In primary school, when pupils feel they receive a higher level of support from their teachers, and when pupil-teacher relations are perceived as being strong, there is a notable increase in wellbeing, self-esteem, a sense of effectiveness, general life satisfaction, positive emotions, executive functions and self-regulation in children [37, 38].

The majority of interventions seeking to improve teacher-pupil interactions by means of educational resources, emotional support resources, specific organizational arrangements in the classroom yielded positive, but modest returns [22, 38]. Organizational support appears to be the most promising component [38]. Furthermore, teachers’ emotional skills and their efforts to manage emotions have received little attention [37]. Teachers perform numerous actions which can be regarded as forms of “care,” and spent large amounts of time with pupils outside of activities strictly related to cognitive learning, particularly in pre-school facilities [37]. Teachers’ capacities for their own emotional management have a positive impact on their sense of self-efficacy and their capacity to recognize children’s emotions, which in turn influences the emotional capabilities of the children.

With regard to relationships among peers (pupil-pupil interactions), positive relations in this sphere are a protective factor against internalizing and externalizing problems, self-harm and suicide, and are conducive to positive emotions, improving feelings of happiness, self-efficacy, optimism and wellbeing. As with pupil-teacher relations, interventions intended to strengthen relations among peers are beneficial when they succeed in developing social skills. Compared with working individually, getting children to work in collaboration have a positive impact on certain forms of cognitive development such as visual discrimination, visual perception and problem-solving, although it does not appear to have any impact on other capacities such as spatial awareness or reading [39].

Regarding relationships among family and ECEC settings, participatory techniques, with activities suggested by parents, or even family activities involving parents and children, may further boost the sense of engagement for family. One review also highlights the reciprocal connections between the environment in ECEC settings and the home environment [40]. It has also been noted that differentiation between the play materials at hand in the home and nursery environments was positively correlated with greater autonomy and the capacity to express positive emotions [40]. Moreover, it has been shown that parents and families are of the utmost importance during the transition to primary school [41].

#### Spatial Environment of Educational Facilities and Structures

The spatial component of educational structures is sometimes described as a “third educator”. Moving away from its former “passive” definition, it is now considered a complex and dynamic reality.

In ECEC settings, the “homely” atmosphere which prevails in facilities for very young children—including the presence of cozy spaces, outdoor spaces, spaces for family interactions and a general attention to aesthetic details—may help to prevent the development of social and emotional problems in children [40]. In one study cited in the same review [40], three-year-old children declare their preference for “soft” colors and elements in cozy spaces, whereas five-year-old children express a preference for “smooth” spaces with lively colors and harder surfaces. A review has also noted the beneficial effects of reading areas, books and writing materials on voluntary learning through play, and the acquisition of cognitive skills [40].

With regard to the outdoor play areas, children of pre-school age are more inclined to engage in functional and dramatic play (more complex forms of play) outdoors than they are indoors [40]. Natural materials and outdoor play areas may boost physical activity and functional, dramatic, independent play, as well as the overall quality of social behaviors. The physical diversity of outdoor facilities helps children to develop motor skills such as balance and coordination. Play areas specifically designed to bring children into contact with the natural environment may encourage independent exploration, autonomous constructive play, dramatic play and scientific experimentation through play, i.e., observing nature [40]. Introducing natural materials to manufactured play areas encourages decision-making, problem-solving, engagement and self-regulation, particularly in open play structures which enable children to test their courage [40]. Mobile physical activity equipment and avoiding overcrowding in play areas allows children to be more physically active [42], to have more complex play interactions and to exhibit less aggressive behavior [40].

In terms of the impact of facility organization, in ECEC settings, striking a balance between the simplicity and complexity of facilities or games may be important when it comes to nurturing interaction and cooperation. For example, a dollhouse can be regarded as a complex toy whereas a push-along toy is simple. Painting walls different colors (complex characteristics) may also encourage cooperation [40]. The balance between opened and closed spaces is also important to modulate interactions and promote engagement with learning activities. While complexity is important to stimulate children, it is also important to clarify the spatial organization of play areas, particularly by creating thematic zones which reinforce the continuity of play for the children, encouraging exploration, interaction and cooperation. Providing healthy food and ready access to water and sanitation facilities in schools can improve the health-related behaviors of children, particularly in terms of reducing the incidence of illness resulting from poor hygiene [42–44].

In conclusion, the co-construction of play areas, where children and teachers collaborate in the design process, has been shown to be essential to better reflect the perceptions and representations of both parties. Indeed, co-construction appear to be a fundamental priority when designing complex systems which define the physical environment of educational facilities [40].

#### Social Inequalities in Educational Facilities and Structures

The educational careers of children between the ages of 0 and 12 often involve changing institutions, particularly if the employment circumstances of their parents change [11]. The consequences of social inequalities may be mitigated by the capacity of educational facilities to provide a counterpoint to “chaotic environments” by establishing routines, imposing regular sleep patterns (nap time) and improving self-regulation through activities tailored to children’s needs [11, 45]. The educational facilities and structures can also reinforce social inequalities [9]: gender and ethnic inequalities are specifically addressed in some reviews included in our scoping of the literature. First, we define how institutions influence gender inequalities, and second, we examine how they influence ethnic inequalities.

Gender inequalities have been explicitly reported in three reviews [39, 46, 47]. For example, discussion and cognitive development are greater for boys with low learning ability when they are in a collaborative task with a girl with high learning ability. Whereas for girls with low learning ability, collaborating with a boy with high learning ability shows no additional advantage over independent work, which may be linked to power imbalances in collaboration [39]. On the other hand, socio-emotional learning is found to have a greater beneficial effect on the mental health of girls than boys, and girl pupils are more sensitive to potential conflicts at the start of the year between students and teachers, and revealed more anxiety about these events [47]. They are also more at risk of developing mental health difficulties than boys, such difficulties being strongly linked to the pressure to achieve good grades at school [47]. Parental involvement in children’s school results, and fear for the future based on school results, have a strong negative influence on girls’ mental health [46]. However, there are still questions about the relationship between mental health and the pressure to get good grades, and about the role of other determinants in this relationship [47]. The fact that educational settings can reinforce gender inequalities is raised in a review that mentions that this element is often posited as a hypothesis but not always analyzed in depth [46].

Ethnic inequalities have been reported in one review [48]. In the United States, it has also been observed that African-Americans suffer from the highest rates of educational exclusion and that boys are at the greatest risk of exclusion [48]. The problem is that exclusion makes pupils miss out on teaching time, as well as relationships and interactions within the establishment, leading to disaffection, negative consequences on the school environment and long-term complications for the excluded pupils, compromising their chances of successfully completing their education and multiplying the risk of failure, dropping out, absenteeism, problematic social behaviors and even, in some cases, hastening their entanglement with the juvenile justice system [48].

## Discussion

### A Conceptual Framework for Action: From Structural to Structuring Determinants of Education

The last section on social inequalities highlights that the educational determinants like education activities, the interpersonal relation within educational institution and? Spatial environment can reproduce social inequalities if social structures are not considered. But the education system also has the capacity to transform the inequalities generated by the structures [48]. For example, modifying the school environment may modify the roles children take on in play. One study observed that, after changing the outdoor play environment, children who had been dominant in the previous playground configuration did not always maintain this dominance following the introduction of greenery and trampolines to the playground [40]. Moreover, the perceived manifestation of diversity policies improves psychological adjustment to school among immigrant youths [48]. A review shows that culturally sensitive mentoring programs for ethnic minority and socially disadvantaged young people can lead to improved mental health [48]. School values must incorporate a degree of sensitivity to other social characteristics and adopt an intersectional perspective to encourage behavior conducive to a productive learning environment, good relations within the school and an all-round positive school environment. Educational systems have the power to transform societies from the inside, creating fairer systems and instilling the values of social justice from early on.

**FIGURE 2:**
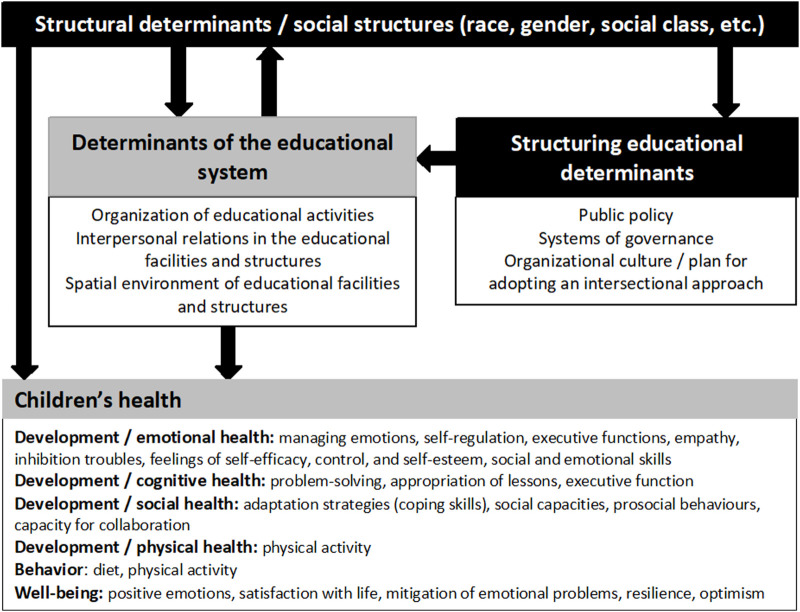
Conceptual framework for action (APPIE study, Bordeaux, France, 2024).

Based on the results contained in this review, our ambition is to develop an active approach to the notion of structural determinants. We thus propose the concept of “structuring determinants” referring to those determinants which shape the effects of social structures (race, gender, social class, etc.) on the health of children in different environments (including educational facilities and structures). The structuring determinants may take the form of policies, cultural influences or systems of governance. In the long term, these structuring determinants may also have a conditioning effect on lived environments, empowering them to alter social structures. Structuring determinants are therefore determinants which serve to mitigate the impact of social structures on social systems (like educational systems). Moreover, the focus on structuring determinants may mitigate the impact of exceptional situations like the COVID-19 successive lockdowns that considerably affected children’s health [49]. By integrating the results identified in our literature review, as well as the contribution made by the concept of structuring determinants, we can construct a more comprehensive framework for understanding the ways in which social structures, lived environments like educational systems and structuring determinants can influence children’s health (Figure 2).

### Limitations and Avenues for Further Research

As with any review, we had certain hypotheses for the type of reviews which would be pertinent to our analysis. We expected to find more reviews dealing with parental involvement and the home environment. However, a large proportion of reviews matching this description were in fact excluded from our selection because they were interventional in their focus but did not consider the effect of such interventions on children’s health. We intentionally excluded meta-analyses, because the aim of this review was to offer a scope of the different educational determinant.

We also excluded a large number of reviews focusing on the inclusion of children on the autism spectrum. We were surprised to find that this subject was approached exclusively from the angle of the inclusive capacities of pupils, rather than the inclusive capacities of schools, which could have been a way of addressing structural determinants. We thus decided to exclude the articles in question because they dealt with a population with special needs. In addition, the design of our study is a scoping review of reviews, which might also explain that we found less publications on this issue. Nevertheless, published studies dealing with autism, public policies and the right to education show that this issue is particularly important [50–52]. Moreover, children with special education needs benefit from inclusive education and schools, as institutions, also benefit from such inclusive approach. The council of Europe reminds that “Inclusive education benefits all learners. It is not limited to integrating children with specific needs into mainstream education, but has a positive impact on all children, the school institutions and the community at large” [53]. A scoping review focused on inclusive education as a whole education approach (with respect to disability, gender, ethnicity, etc.) and its impact on children health could shed further light on this relevant topic.

We also had to exclude some reviews about school workforce because the link with children health was not explicitly addressed. This is the reason why this issue only appears in the section about the interpersonal relations at school. However, it remains a crucial issue. Structuring determinants like public policies may focus on the importance of the quality of teachers’ initial and continuing education training. Furthermore, a greater attention should be placed on teachers’ working conditions as well as that of the entire education community, because such conditions may have an impact both on teachers and students. For example, it has been demonstrated that pupils taught by teachers who feel that their working conditions are unfair are more likely to be dissatisfied at school, to play truant, to experience psychosomatic troubles and depression, and to receive lower grades than those taught by teachers not burdened by this feeling of injustice [54].

As noted above, it would be pertinent to build upon this initial review with a follow-up focusing on structuring determinants within educational systems which are conducive to the wellbeing of children.

### Conclusion

We observed four main categories of determinants (the organization of educational activities, the interpersonal relations in the education facilities and structures and spatial environment in the educational facilities and structures) which influence children’s health. The section on social inequalities has allowed us to make a distinction between structural and structuring determinant and to propose a comprehensive framework addressing structural inequalities in educational systems.
