# Supplementary material for: The Influence of Educational Determinants on Children’s Health: A Scoping Review of Reviews
Source: Public Health Rev. 2024 Jun 5;45:1606372. doi: 10.3389/phrs.2024.1606372 (PMC11188304; doi:10.3389/phrs.2024.1606372)
Supplement: Supplementary file 1 [file Table1.docx]

| Concept | EBSCOHOST | Web of sciences | PubMed | PROQUEST |
| --- | --- | --- | --- | --- |
| School / institutionalized education | “child* N2 education” or “early N2 education” or “curriculum” or “extracurricular” or “educational N2 activit*” or “classroom” or “school*” or “academic institution*” or “education* institution*” or “playground” or “nuser*” or "kindergarten" or "kinder-garten" or “day care “ or “daycare” or “day-care” or “child care” or “child care “ or “child-care” | “child* SAME education*” or “early SAME education*” or “curriculum” or “extracurricular” or “education* SAME activit*” or “classroom” or “school*” or “academic institution*” or “education* institution*” or “playground” or “nuser*” or "kindergarten" or "kinder-garten" or “day care “ or “daycare” or “day-care” or “child care” or “child care “ or “child-care” | ***Mots-clés***  “Education” or “learning” | "child* NEAR/2 education*" OR “early NEAR/2 education*” OR “curriculum” or “extracurricular” or “education* NEAR/2 activit*” or “classroom” OR "learning*" OR "school*" OR "academic institution*" OR “education* institution*” OR “playground” OR "nuser*" OR "kindergarden" OR "kinder-garden" OR "day care " OR "daycare" OR "day-care" OR "child care" OR "child care " OR "child-care" |
| Child health | ***Mots-clés***  “Child health” / “CHILDREN'S health”  ***OU***  ***Mots dans résumé / Titre***  "child*" or "infant*" or "baby" or “babies” or "pre-schooler*" or “preschooler*” or "toddler*"  à proximité de (N2)  “health” or “development” or “skills” or “welfare” or “well being” or “wellbeing” or “well-being” | "child*" or "infant*" or "baby" or “babies” or "pre-schooler*" or “preschooler*” or "toddler*"  à proximité de (NEAR/2)  “health” or “development” or “skills” or “welfare” or “well being” or “wellbeing” or “well-being”  ***OU***  "child*" or "infant*" or "baby" or “babies” or "pre-schooler*" or “preschooler*” or "toddler*"  ET  “cognitive development" or "emotion* development" or "motor development" or “socio* development” or "psych* development" or "cognitive skills" or "emotion* skills" or "motor skills" or “socio* skills” or "psych* skills" | ***Mots-clés***  “Child health”  ***OU***  ***Mots dans résumé / Titre***  "child*" or "infant*" or "baby" or “babies” or "pre-schooler*" or “preschooler*” or "toddler*" | "health" or "development" or “skills” or "welfare" or “well-being” or “well being” or “wellbeing” |
|  |  |  | “health” or “development” or “skills” or “welfare” or “well being” or “wellbeing” or “well-being | "child*" or "infant*" or "baby" or “babies” or "pre-schooler*" or “preschooler*” or "toddler*" |
| Review | “Systematic review” or “scoping review” or “meta analysis” or “literature N2 review” or “umbrella review” or “review of reviews” or “systematic review of reviews” or “review of systematic reviews” or “overviews of reviews” or “summary of systematic reviews” or “summary of reviews” or “synthesis of reviews” or "meta-synthesis" or "meta-research" | “Systematic review” or “scoping review” or “meta analysis” or “literature SAME review” or “umbrella review” or “review of reviews” or “systematic review of reviews” or “review of systematic reviews” or “overviews of reviews” or “summary of systematic reviews” or “summary of reviews” or “synthesis of reviews” or "meta-synthesis" or "meta-research" | ***Publication type***  “Systematic review” or “scoping review” or “umbrella review” or “review of reviews” or “systematic review of reviews” or “review of systematic reviews” or “overviews of reviews” or “summary of systematic reviews” or “summary of reviews” or “synthesis of reviews” or "meta-synthesis" or "meta-research" | “Systematic review” or “scoping review” or “literature NEAR/2 review” or “umbrella review” or “review of reviews” or “systematic review of reviews” or “review of systematic reviews” or “overviews of reviews” or “summary of systematic reviews” or “summary of reviews” or “synthesis of reviews” or "meta-synthesis" or "meta-research" |
