# Supplementary material for: The Influence of Educational Determinants on Children’s Health: A Scoping Review of Reviews
Source: Public Health Rev. 2024 Jun 5;45:1606372. doi: 10.3389/phrs.2024.1606372 (PMC11188304; doi:10.3389/phrs.2024.1606372)
Supplement: Supplementary file 2 [file Table2.docx]

| Syntaxes |
| --- |
| Databases |
| EBSCOHOST (APA PsycInfo, APA PsycArticles, Psychology and Behavioral Sciences Collection, SocINDEX with Full Text, CINAHL Complete) |
| (AB (“child* N2 education” or “early N2 education” or “curriculum” or “extracurricular” or “educational N2 activit*” or “school*” or “academic institution*” or “education* institution*” or “playground” or “nuser*” or "kindergarten" or "kinder-garten" or "kindergarden" or "kinder-garden" or “day care “ or “daycare” or “day-care” or “child care” or “childcare “ or “child-care”))  AND ((AB ("child* N2 health" or “child* N2 development” or “child* N2 skills” or “child* welfare” or “child* wellbeing" or “child* well-being” or “child* well being” or "infant* NEAR/2 health" or “infant* N2 development” or “infant* N2 skills” or “infant* welfare” or “infant* wellbeing" or “infant* well-being” or “infant* well being” or "baby N2 health" or “baby N2 development” or “baby N2 skills” or “baby welfare” or “baby wellbeing" or “baby well-being” or “baby well being” or "babies NEAR/2 health" or “babies N2 development” or “babies N2 skills” or “babies welfare” or “babies wellbeing" or “babies well-being” or “babies well being” or "pre-schooler* N2 health" or “pre-schooler* NEAR/2 development” or “pre-schooler* N2 skills” or “pre-schooler* welfare” or “pre-schooler* wellbeing" or “pre-schooler* well-being” or “pre-schooler* well being” or "preschooler* N2 health" or “preschooler* N2 development” or “preschooler* N2 skills” or “preschooler* welfare” or “preschooler* wellbeing" or “preschooler* well-being” or “preschooler* well being” or "toddler* N2 health" or “toddler* N2 development” or “toddler* N2 skills” or “toddler* welfare” or “toddler* wellbeing" or “toddler* well-being” or “toddler* well being” or "child* N2 cognitive development" or “child* N2 emotion* development” or “child* N2 motor development” or “child* soci* development” or “child* psycho* development" or “child* cognitive skills” or “child* emotion* skills” or “child* motor skills” or “child* soci* skills” or “child* psych* skills” or "infant* N2 cognitive development" or “infant* N2 emotion* development” or “infant* N2 motor development” or “infant* soci* development” or “infant* psycho* development" or “infant* cognitive skills” or “infant* emotion* skills” or “infant* motor skills” or “infant* soci* skills” or “infant* psych* skills” or "baby N2 cognitive development" or “baby N2 emotion* development” or “baby N2 motor development” or “baby soci* development” or “baby psycho* development" or “baby cognitive skills” or “baby emotion* skills” or “baby motor skills” or “baby soci* skills” or “baby psych* skills” or "babies N2 cognitive development" or “babies N2 emotion* development” or “babies N2 motor development” or “babies soci* development” or “babies psycho* development" or “babies cognitive skills” or “babies emotion* skills” or “babies motor skills” or “babies soci* skills” or “babies psych* skills” or "pre-schooler* N2 cognitive development" or “pre-schooler* N2 emotion* development” or “pre-schooler* N2 motor development” or “pre-schooler* soci* development” or “pre-schooler* psycho* development" or “pre-schooler* cognitive skills” or “pre-schooler* emotion* skills” or “pre-schooler* motor skills” or “pre-schooler* soci* skills” or “pre-schooler* psych* skills” or "preschooler* N2 cognitive development" or “preschooler* N2 emotion* development” or “preschooler* N2 motor development” or “preschooler* soci* development” or “preschooler* psycho* development" or “preschooler* cognitive skills” or “preschooler* emotion* skills” or “preschooler* motor skills” or “preschooler* soci* skills” or “preschooler* psych* skills” or "toddler* N2 cognitive development" or “toddler* N2 emotion* development” or “toddler* N2 motor development” or “toddler* soci* development” or “toddler* psycho* development" or “toddler* cognitive skills” or “toddler* emotion* skills” or “toddler* motor skills” or “toddler* soci* skills” or “toddler* psych* skills”)) OR ((SU CHILDREN'S health) OR (SU Child Health)))  AND (AB (“Systematic Review” or “meta-analysis” or “scoping review” or “literature N2 review” or “umbrella review" or “review of reviews” or “systematic review of reviews” or “review of systematic reviews” or “overviews of reviews” or “summary of systematic reviews” or “summary of reviews” or “synthesis of reviews” or "meta-synthesis" or "meta-research")) |
| WEB OF SCIENCES |
| (TS=(child* NEAR/2 education* or early NEAR/2 education* or “curriculum” or “extracurricular” or education* NEAR/2 activit* or “school*” or “academic institution*” or “education* institution*” or “playground” or “nuser*” or "kindergarten" or "kinder-garten" or "kindergarden" or "kinder-garden" or “day care “ or “daycare” or “day-care” or “child care” or “childcare “ or “child-care”))  AND  (TS=(child* NEAR/2 health or child* NEAR/2 development or child* NEAR/2 skills or child* welfare or child* wellbeing or “child* well-being” or “child* well being” or infant** NEAR/2 health or infant NEAR/2 development or infant* NEAR/2 skills or “infant* welfare” or “infant* wellbeing" or “infant* well-being” or “infant* well being” or baby NEAR/2 health or baby NEAR/2 development or baby NEAR/2 skills or “baby welfare” or “baby wellbeing" or “baby well-being” or “baby well being” or babies NEAR/2 health or babies NEAR/2 development or babies NEAR/2 skills or “babies welfare” or “babies wellbeing" or “babies well-being” or “babies well being” or pre-schooler* NEAR/2 health or pre-schooler* NEAR/2 development or pre-schooler* NEAR/2 skills or “pre-schooler* welfare” or “pre-schooler* wellbeing" or “pre-schooler* well-being” or “pre-schooler* well being” or preschooler* NEAR/2 health or preschooler* NEAR/2 development or preschooler* NEAR/2 skills or “preschooler* welfare” or “preschooler* wellbeing" or “preschooler* well-being” or “preschooler* well being” or toddler* NEAR/2 health or toddler* NEAR/2 development or toddler* NEAR/2 skills or “toddler* welfare” or “toddler* wellbeing" or “toddler* well-being” or “toddler* well being” or "child* NEAR/2 cognitive development" or “child* NEAR/2 emotion* development” or “child* NEAR/2 motor development” or “child* soci* development” or “child* psycho* development" or “child* cognitive skills” or “child* emotion* skills” or “child* motor skills” or “child* soci* skills” or “child* psych* skills” or "infant* NEAR/2 cognitive development" or “infant* NEAR/2 emotion* development” or “infant* NEAR/2 motor development” or “infant* soci* development” or “infant* psycho* development" or “infant* cognitive skills” or “infant* emotion* skills” or “infant* motor skills” or “infant* soci* skills” or “infant* psych* skills” or "baby NEAR/2 cognitive development" or “baby NEAR/2 emotion* development” or “baby NEAR/2 motor development” or “baby soci* development” or “baby psycho* development" or “baby cognitive skills” or “baby emotion* skills” or “baby motor skills” or “baby soci* skills” or “baby psych* skills” or "babies NEAR/2 cognitive development" or “babies NEAR/2 emotion* development” or “babies NEAR/2 motor development” or “babies soci* development” or “babies psycho* development" or “babies cognitive skills” or “babies emotion* skills” or “babies motor skills” or “babies soci* skills” or “babies psych* skills” or "pre-schooler* NEAR/2 cognitive development" or “pre-schooler* NEAR/2 emotion* development” or “pre-schooler* NEAR/2 motor development” or “pre-schooler* soci* development” or “pre-schooler* psycho* development" or “pre-schooler* cognitive skills” or “pre-schooler* emotion* skills” or “pre-schooler* motor skills” or “pre-schooler* soci* skills” or “pre-schooler* psych* skills” or "preschooler* NEAR/2 cognitive development" or “preschooler* NEAR/2 emotion* development” or “preschooler* NEAR/2 motor development” or “preschooler* soci* development” or “preschooler* psycho* development" or “preschooler* cognitive skills” or “preschooler* emotion* skills” or “preschooler* motor skills” or “preschooler* soci* skills” or “preschooler* psych* skills” or "toddler* NEAR/2 cognitive development" or “toddler* NEAR/2 emotion* development” or “toddler* NEAR/2 motor development” or “toddler* soci* development” or “toddler* psycho* development" or “toddler* cognitive skills” or “toddler* emotion* skills” or “toddler* motor skills” or “toddler* soci* skills” or “toddler* psych* skills”))  AND (TS= (“Systematic Review” or “meta-analysis” or “scoping review” or “literature N2 review” or “umbrella review" or “review of reviews” or “systematic review of reviews” or “review of systematic reviews” or “overviews of reviews” or “summary of systematic reviews” or “summary of reviews” or “synthesis of reviews” or "meta-synthesis" or "meta-research")) |
| PUBMED |
| (("learning"[MeSH Terms] OR "leisure activities"[MeSH Terms] OR ("educational status"[MeSH Terms] OR "education"[MeSH Terms])) AND ((("child*"[Title/Abstract] OR "infant*"[Title/Abstract] OR "baby"[Title/Abstract] OR "babies"[Title/Abstract] OR "pre schooler*"[Title/Abstract] OR "preschooler*"[Title/Abstract] OR "toddler*"[Title/Abstract]) AND ("health"[Title/Abstract] OR "development"[Title/Abstract] OR "skills"[Title/Abstract] OR "welfare"[Title/Abstract] OR "well-being"[Title/Abstract] OR "well-being"[Title/Abstract] OR "wellbeing"[Title/Abstract])) OR "child health"[MeSH Terms]) AND (newborn[Filter] OR allinfant[Filter] OR infant[Filter] OR preschoolchild[Filter] OR child[Filter])) AND ("Systematic Review"[title/abstract] or "scoping review"[title/abstract] or "literature review"[title/abstract] or "review of the literature"[title/abstract] or "umbrella review"[title/abstract] or "review of reviews"[title/abstract] or "systematic review of reviews"[title/abstract] or "review of systematic reviews"[title/abstract] or "overviews of reviews"[title/abstract] or "summary of systematic reviews"[title/abstract] or "summary of reviews"[title/abstract] or "synthesis of reviews"[title/abstract] or "meta-synthesis"[title/abstract] or "meta-research"[title/abstract]) |
| PROQUEST (ERIC, International Bibliography of the Social Sciences (IBSS)‎, Linguistics and Language Behavior Abstracts (LLBA)‎, Political Science Database, Sociological abstract) |
| ab("child* NEAR/2 education*" OR “early NEAR/2 education*” OR “curriculum” or “extracurricular” or “education* NEAR/2 activit*” or “classroom” OR "learning*" OR "school*" OR "academic institution" OR "academic institutions" OR “education* institution*” OR “playground” OR "nuser*" OR "kindergarden" OR "kinder-garden" OR "day care " OR "daycare" OR "day-care" OR "child care" OR "child care " OR "child-care") AND ab("health" OR "development" OR "skills" OR "welfare" OR "well-being" OR "well being" OR "wellbeing") AND ab("child*" OR "infant*" OR "baby" OR "babies" OR "pre-schooler*" OR "preschooler*" OR "toddler*") AND ab(“Systematic Review” or “scoping review” or “literature NEAR/2 review” or “umbrella review" or “review of reviews” or “systematic review of reviews” or “review of systematic reviews” or “overviews of reviews” or “summary of systematic reviews” or “summary of reviews” or “synthesis of reviews” or "meta-synthesis" or "meta-research") |

| Grey litterature |  |
| --- | --- |
| OCDE | Child health and education |
| British Education index |  |
| Center for the developing child (Harvard) |  |
| UNESCO |  |
| EURYCIDE |  |
| OMS |  |
