# Supplementary material for: The Influence of Educational Determinants on Children’s Health: A Scoping Review of Reviews
Source: Public Health Rev. 2024 Jun 5;45:1606372. doi: 10.3389/phrs.2024.1606372 (PMC11188304; doi:10.3389/phrs.2024.1606372)
Supplement: Supplementary file 3 [file Table3.docx]

i) citation details, ii) objectives of the review, iii) type of review, iv) details regarding participants, v) setting and context vi) number of databases sourced and searched, vii) date range of database searching, viii) publication date xx) range of studies included in the review that inform each outcome of interest, ix) number of studies, types of studies and country of origin of studies included in each review, x) instrument used to appraise the primary studies and the rating of their quality, xi) outcomes reported that are relevant to the umbrella review question, xii) method of synthesis/analysis used to synthesize the evidence and xiii) comments or notes the scoping review authors may have regarding any of the studies thus included, xiv) the discipline of the review, xv) type of methodology, xvi) the health outcomes reported, xvii) the educational outcomes reported
