# Supplementary material for: The Influence of Educational Determinants on Children’s Health: A Scoping Review of Reviews
Source: Public Health Rev. 2024 Jun 5;45:1606372. doi: 10.3389/phrs.2024.1606372 (PMC11188304; doi:10.3389/phrs.2024.1606372)
Supplement: Supplementary file 4 [file Table4.docx]

| **Title** | **Author** | **Abstract** | **Date** | **Review** |  |
| --- | --- | --- | --- | --- | --- |
| 1. Organization and structure of educational activities (in general) | | | | | |
| School educational models and child mental health among K-12 students: a scoping review | Yu et al. | This review sought to provide a comprehensive understanding of key factors involved in models of school education contributing to student mental health development, interrelationships among these factors and the cross-cultural differences across nations and societies. Based on the five key factors (including curriculum, homework and tests, physical activities, interpersonal relationships and after-school activities) identified in student mental development according to thematic analyses, a multi-component school educational model integrating academic, social and physical factors was proposed so as to conceptualize the five school-based dimensions for K-12 students to promote student mental health development. | 2022 | Child and Adolescent Psychiatry and Mental Health | Early childhood education and care setting / primary school / secondary school |
| Social and Emotional Learning | | | | | |
| Identifying Common Practice Elements to Improve Social, Emotional, and Behavioral Outcomes of Young Children in Early Childhood Classrooms. | McLeod et al. | The purpose of this paper is to report findings from a study designed to identify common practice elements found in comprehensive intervention models (i.e., manualized interventions that include a number of components) or discrete practices (i.e., a specific behavior or action) designed to target social, emotional, and behavioral learning of young children who exhibit problem behavior. Twenty-four practice elements were identified and classified into content (the goal or general principle that guides a practice element) and delivery (the way in which a teacher provides instruction to the child) categories. | 2017 | Prevention Science | Early childhood education and care setting |
| Social and Emotional Learning in Preschool Settings: A Systematic Map of Systematic Reviews | Djamnezhad et al. | Social and emotional learning (SEL) programs in early childhood education and care (ECEC) have gained an increased interest due to its potential to improve child health and educational outcomes. We aimed to identify existing systematic reviews on universal, curriculum-based SEL interventions in ECEC settings (children aged 0 to 7 years), assess their risk of bias, synthesize the findings and identify knowledge gaps. SEL interventions in preschool settings must be considered knowledge gaps. There is a need for more high-quality primary studies and further systematic reviews that adhere to strict scientific methods and address the overwhelming heterogeneity in field, in terms of interventions, settings and outcomes. | 2021 | Frontiers in education | Early childhood education and care setting |
| Mindfulness interventions | | | | | |
| Scoping Review of Yoga in Schools: Mental Health and Cognitive Outcomes in Both Neurotypical and Neurodiverse Youth Populations | Hart et al. | Therefore, the aim of this scoping review is to understand what is known about the relationships between yoga in schools and mental health and cognition in neurotypical and neurodiverse youth populations. Substantial evidence to support the use of school-based yoga programmes for the improvement of anxiety, self-concept, resilience, depression, self-esteem, subjective and psychological well-being, executive function, inhibition, working memory, attention and academic performance in neurotypical populations was found. Evidence to support school-based yoga programmes in neurodiverse populations with improvements in self-concept, subjective well-being, executive function, academic performance and attention was also found. | 2022 | Children | Early childhood education and care setting / primary school / secondary school |
| Yoga and Mindfulness Interventions for Preschool-Aged Children in Educational Settings: A Systematic Review | Sun et al. | A systematic review using PRISMA guidelines was undertaken to explore the effect of YMP on social emotional function among preschool-aged children (3-5 years). Programs appeared to produce better results when implemented for at least 6 weeks and among children who had lower baseline social-emotional functioning. YMP constitute a promising strategy for social emotional development in early childhood settings, but additional rigorously designed studies are needed to expand understanding of how and why these programs are effective. | 2021 | International Journal of Environmental Research and Public Health | Early childhood education and care setting |
| Influence of Mindfulness-Based Interventions on Executive Functions in Childhood: A Systematic Review | Jansen et al. | The present review seeks to describe and compare MBIs characteristics, assessment methods, and effects in youth between 3 and 18 years old considering four developmental periods. Results show that the frequency of the sessions and program duration varies widely. Differences were observed in instructors’ training and in assessment strategies. Discrepancies were observed regarding the effects of MBIs both within and between periods in cognitive, socio-emotional, symptoms, and mindfulness variables. Consistency was observed in prosocial behaviors for preschoolers, and in emotional and behavioral problems and hyperactivity in ages between preschool and early adolescence. Nevertheless, it was impossible to compare most results and determine consistency or discrepancy due to the lack of studies. | 2016 | Zeitschrift fur entwicklungspsychology und padagogische psychologie | Early childhood education and care setting / primary school / secondary school |
| Innovative educational formats | | | | | |
| Getting Out of the Classroom and Into Nature: A Systematic Review of Nature-Specific Outdoor Learning on School Children's Learning and Development | Mann et al. | The aim of the review was to undertake a narrative synthesis of international evidence of nature-specific outdoor learning and its benefits for personal and social development, wellbeing and academic progress. The reported benefits of learning in natural outdoor settings include: increased student engagement and ownership of their learning, some evidence of academic improvement, development of social and collaborative skills, and improved self-concept factors. Nature-specific outdoor learning has measurable socio-emotional, academic and wellbeing benefits, and should be incorporated into every child's school experience with reference to their local context. Teacher pre-service and in-service education needs to include a focus on how natural settings can be used effectively for learning. | 2022 | Front Public Health | Early childhood education and care setting / primary school / secondary school |
| The impact of school gardens on youth social and emotional learning: a scoping review | Lohr et al. | Social and Emotional Learning (SEL) has five competencies: relationship skills, responsible decision-making, self-awareness, social awareness, and self-management. A promising practice to promote positive SEL is school garden programming. There is a need to understand how school gardens impact SEL by consolidating existing research. While the included qualitative studies demonstrated that school garden programming can positively influence SEL, the included quantitative studies had few statistically significant results. Thus, at this time we can only say that qualitative research from five studies suggests that school garden programs have the potential to successfully enhance experiences that promote SEL but more research is needed to further investigate this claim. | 2021 | Journal of adventure education and outdoor learning | Primary school |
| Impact of School Garden Participation on the Health Behaviors of Children | Schneider et al. | This systematic review examined the impact of garden-based intervention on nutritional knowledge, fruit/vegetable consumption, taste preferences, physical activity, and math/ science academic achievements. Findings suggest that school gardening programs ideally should include a nutritional component to increase participants' nutritional knowledge, and fruit and vegetable consumption, as well as broaden taste preferences. Conclusion: An educational curriculum in addition to gardening activities appears to be an effective strategy for enhancing attitudes toward healthy foods and healthy dietary behaviors. | 2017 | Health behavior and policy review | Primary school |
| Garden-based interventions and early childhood health: an umbrella review | Skelton et al. | The authors performed an umbrella review of garden-based interventions to examine their role in early childhood health promotion for children ages 6 years and younger, examining effective components of garden-based interventions and critically evaluating existing evidence. Across reviews, garden based-interventions were most effective at improving nutrition-related outcomes for children, including nutritional status and fruit and vegetable consumption. Few reviews examined child health outcomes of garden-based interventions that were not nutrition related, such as physical activity, or academic performance. Across settings, there was the most evidence in support of garden-based interventions conducted in home gardens, compared to evidence from early care and education or community settings. We were unable to report on most effective components of garden-based interventions due to limitations of included reviews. | 2020 | International Journal of Behavioral Nutrition and Physical Activity | Early childhood education and care setting |
| Children's literature to promote students' global development and wellbeing | Pulimeno et al. | In this paper, we carried out a review to examine pedagogic, didactic and psychological/therapeutic dimensions of children’s literature, with the aim of highlighting its role in promoting students’ holistic development and wellbeing. From a pedagogic point of view, tales convey basic values useful for children lives. In a didactic perspective, properly chosen storybooks represent a valuable resource for school activities, improving students’ language skills and building up a friendly/respectful classroom environment. Children stories are also used by health professionals for therapeutic purposes (bibliotherapy) to prevent unhealthy habits and addictions, or address psychosomatic disorders. Finally, storybooks and web-based/digital stories can be an effective vehicle for health contents, to encourage the adoption of healthy lifestyles among schoolchildren. | 2020 | Health promotion perspectives | Primary schools |
| Extra-curricular activities | | | | | |
| School-related sedentary behaviours and indicators of health and well-being among children and youth: a systematic review | Kuzik et al. | The purpose of this systematic review was to examine the associations between school-related sedentary behaviours and indicators of health and well-being in children and youth (~ 5–18 years) attending school. Active lessons were favourable (72%), compared to more school-related sedentary behaviours, when examining associations for all health and well-being indicators. More homework was favourable across all health and well-being indicators in 4% of extracted associations for primary school children, and 25% of extracted associations for secondary school children. However, ≥2 h/day of homework appeared to be unfavourable for health and well-being. Limitations for synthesized studies included generally low quality of evidence and a lack of studies in South American, African, or low-middle income countries. | 2022 | International journal of behavioral nutrition and physical activity | Primary school / secondary school |
| Do School-Based Interventions Focusing on Physical Activity, Fitness, or Fundamental Movement Skill Competency Produce a Sustained Impact in These Outcomes in Children and Adolescents? A Systematic Review of Follow-Up Studies | Lai et al. | The aim of this systematic review was to determine whether typically developing children and adolescents (aged 3–18 years) who have participated in school-based interventions have sustained outcomes in physical activity, fitness, and/ or fundamental movement skill. Three-quarters (ten of 13) of the studies addressing physical activity, reported physical activity behavior change maintenance. The length of follow-up ranged from 6 months to 20 years, and the degree of PA difference reported was between 3 and 14 min per day. Only one of the three studies assessing fitness reported a sustained impact, whilst both studies that assessed fundamental movement skill reported maintenance of effects. | 2014 |  | Early childhood education and care setting / primary school / secondary school |
| Understanding the Benefits of Brief Classroom-Based Physical Activity Interventions on Primary School-Aged Children's Enjoyment and Subjective Wellbeing: A Systematic Review | Papadopoulos et al. | We conducted a systematic review to investigate the effect of brief classroom-based PA interventions on primary school children's subjective wellbeing and enjoyment of physical activity. Thirteen studies examined the effect of brief classroom-based PA interventions on subjective wellbeing. Four found a significant positive effect for children in the intervention group (specifically for children's self-efficacy in learning with video exercises, quality of life, and self-confidence). Eight studies examined the effect of brief classroom-based PA interventions on enjoyment of PA. Five studies reported a positive effect for children in the intervention group and only 1 study found a negative effect for 1 grade level of children in the intervention group. The remaining studies with wellbeing and/or enjoyment outcomes reported no significant effect. Findings from these studies indicate that the integration of brief PA breaks may support PA enjoyment and specific self-beliefs and quality of life perceptions that contribute to children's subjective wellbeing. However, more research is needed to confirm this effect. |  |  | Primary school |
| Informal STEM Learning for Young Children: A Systematic Literature Review | Alexandre et al. | This systematic review serves two purposes: to explore the impact of informal STEM learning (ISL) on school readiness and to examine the relationship between ISL and children’s social-emotional development, particularly in children who are dual language learners. The findings illuminate the important role of parents and/or caregivers and the quality of design and interventions used at ISL sites, such as museums and zoos, on how ISL can impact school readiness for preschoolers. No research was found to specifically address the impact of ISL on school readiness for children who are dual language learners. The implications from the findings suggest that further research is needed on ISL for young children, particularly considering the dearth in research on young dual language learners. The implications further suggest that parents, ISL site designers, facilitators, and educators can benefit from learning about the impact of ISL on school readiness. | 2022 | International Journal of Environmental Research and Public Health | Early childhood education and care setting / primary school |
| 1. The interpersonal relations | | | | | |
| Early Childhood Teachers as Socializers of Young Children's Emotional Competence | Denham et al. | Young children’s emotional competence—regulation of emotional expressiveness and experience when necessary, and knowledge of their own and other’s emotions—is crucial for social and academic (i.e., school) success. Thus, it is important to understand the mechanisms of how young children develop emotional competence. Both parents and teachers are considered as important socializers of emotion, providing children experiences that promote or deter the development of emotional competence. However, compared to parents, early childhood teachers’ roles in socializing young children’s emotional competence have not been examined. Based on the findings from research on parental socialization of emotion, in this theoretical review we explore possible teacher roles in the development of young children’s emotional competence. Additionally, we suggest future research focusing on early childhood teacher socialization of emotion, and discuss theoretical and practical benefits of such research. | 2012 | Early childhood education journal | Early childhood education and care setting |
| The role of collaboration in the cognitive development of young children: a systematic review | Sills et al. | Collaboration is a key facilitator of cognitive development in early childhood; this review evaluates which factors mediate the impact of collaborative interactions on cognitive development in children aged 4-7 years. Immediate benefits of collaboration on cognitive development are highlighted for same-age peers. Collaborative interactions are beneficial for tasks measuring visual perception, problem-solving and rule-based thinking, but not for word-reading and spatial perspective-taking. Collaboration is particularly beneficial for lower-ability children when there is an ability asymmetry. High-ability children either regressed or did not benefit when paired with lower-ability participants. | 2016 | Child care health and development | Early childhood education and care setting |
| Strengthening Executive Function and Self-Regulation Through Teacher-Student Interaction in Preschool and Primary School Children: A Systematic Review | Sankalaite et al. | Executive functions (EF) and self-regulation (SR) are fundamental for children's learning, school functioning and academic achievement. Recent research has shifted the attention towards malleable environmental factors; more specifically, to the role of school and classroom environment as an important developmental context for promoting children's EF/SR skills and, in turn, their cognition and behaviour. Numerous observational studies have shown a correlation between the quality of teacher-student relationship (TSR) at the dyadic level or teacher-student interaction (TSI) at the classroom level and children's EF/SR skills. The results from 18 included studies indicated that children in treatment groups show higher gains, albeit small-sized, in EF/SR performance compared to controls. Furthermore, TSI manipulation seemed to affect children's SR skills more strongly than children's EF skills. More importantly, the findings revealed the largest effects of these manipulations in children considered vulnerable or disadvantaged, suggesting that the cognitive deficits can be minimised if these children are supported appropriately. | 2021 | Frontiers in psychology | Early childhood education and care setting / primary school |
| Systematic review of interventions in the childcare setting with direct parental involvement: effectiveness on child weight status and energy balance-related behaviours | Van de Kolk et al. | The aim of this systematic review is to evaluate the effectiveness of these interventions with direct parental involvement on the children’s weight status and behavioural outcomes. Childcare-based interventions with direct parental involvement show promising effects on the children’s energy balance-related behaviours. However, evidence on effectiveness is limited, particularly for weight-related outcomes. Better understanding of how to reach and involve parents may be essential for strengthening intervention effectiveness. | 2019 | International Journal of Behavioral Nutrition and Physical Activity | Early childhood education and care setting |
| Interventions targeting motor skills in pre-school-aged children with direct or indirect parent engagement: a systematic review and narrative synthesis | Stevenson et al. | This review aimed to evaluate the effectiveness of motor skill interventions for preschoolers with the inclusion of a parental/home component. Literature searches were completed in PubMed, Sports Discuss and the Cochrane Library. Studies that implemented an intervention with direct or indirect parental engagement and evaluated fundamental movement skill as an outcome measure were included. Seventeen studies met the inclusion criteria and a narrative synthesis of results suggested type of parent engagement may be associated with improvements in preschoolers' motor skills. It can be recommended that early years' motor skill interventions should include parents, ideally encouraging their active participation which may be the most influential form of parent engagement. | 2022 | International Journal of Primary, Elementary and Early Years Education | Early childhood education and care setting |
| Young Children's Health and Wellbeing Across the Transition to School: A Critical Interpretive Synthesis. | Fane et al. | This paper reports on the systematic search and review of the literature relating to the health and wellbeing of young children across the transition to school. The findings are presented in seven thematic categories: current conceptualisations of health and wellbeing, assessment and measurement, ‘school readiness’, service integration, transition actors, ‘at risk’ children, and child voice. These findings illustrate the ways in which concepts have been constructed, identified, and operationalised in early years research, practice, and policy. Moreover, it highlights that ‘what is known’ can be used to inform the review or implementation of services, practices, and partnerships that support child health and wellbeing during the transition to school. | 2016 | Children Australia | Early childhood education and care setting / primary school |
| 1. Spatial environment of educational facilities and structures | | | | | |
| Early Childhood Education and Care Physical Environment and Child Development: State of the art and Reflections on Future Orientations and Methodologies | Berti et al. | In relation to the growing attention to the quality of physical space in early childhood education and care (ECEC), the present scoping review aims to define the state of the art regarding the relationship between the physical environment of ECEC services and the psychological development of children. The contents of the studies identified refer to two main thematic areas: “perception of physical environment,” which includes both children’s and adults’ perceptions, and “relation between physical environment and child development,” which includes behavioral, cognitive, and emotional aspects of development. Although the studies included have used several methods and investigated different aspects of environment, systematic analysis has enabled the identification of some topics that recur among the studies, such as the significance of adults’ awareness about their perception of space, the importance of a child-centered approach, and the relevance of the participation of stakeholders in the design processes. Finally, these main findings are summarized and reflections on future conceptual and methodological orientations in the field are discussed. | 2019 | Educational psychology review | Early childhood education and care setting |
| Which Meso-Level Characteristics of Early Childhood Education and Care Centers Are Associated with Health, Health Behavior, and Well-Being of Young Children? Findings of a Scoping Review | Herr et al. | This scoping review aims to provide an overview of the association between meso-level characteristics (MLCs) of ECEC centers with children’s health, health behavior, and wellbeing. Five categories of MLCs were identified: (1) structural characteristics, (2) equipment/furnishings, (3) location, (4) facilities/environment, (5) culture/activities/policies/practices, and 6) staff. Only very few studies found an association of MLCs with body weight/obesity, and general health and wellbeing. Especially physical activity and mental health were related to MLCs. In general, the location (rural vs. urban, neighborhood status) seemed to be a relevant health aspect. MLCs of ECEC centers appeared relevant for child health indicators to different degrees. Future research should focus on these associations, in detail, to identify concrete ECEC indicators that can support health promotion in early childhood. | 2021 | International Journal of Environmental Research and Public Health | Early childhood education and care setting |
| The Impact of Canadian School Food Programs on Children's Nutrition and Health: A Systematic Review | Colley et al. | This systematic review synthesizes current academic literature pertaining to school nutrition programs in Canada to identify existing interventions and their impacts on children's nutritional knowledge, dietary behaviour, and food intake. The programs incorporated a variety of intervention components including policy, education, family and community involvement, and/or food provision. These multi-component interventions were positively associated with children's development of nutrition knowledge, dietary behaviour changes, and intake of healthy foods; however, barriers associated with intervention duration, intensity, and availability of resources may have influenced the extent to which these programs impacted children's diets and overall health. | 2019 | Canadian Journal of Dietetic Practice and Research | Primary school |
| Water and sanitation in schools: a systematic review of the health and educational outcomes | Jasper et al. | The goal was to characterize the impacts of water and sanitation inadequacies in the academic environment. Chosen studies were divided into six fields based on their specific foci: water for drinking, water for handwashing, water for drinking and handwashing, water for sanitation, sanitation for menstruation and combined water and sanitation. The studies provide evidence for an increase in water intake with increased provision of water and increased access to water facilities. Articles also report an increase in absenteeism from schools in developing countries during menses due to inadequate sanitation facilities. Lastly, there is a reported decrease in diarrheal and gastrointestinal diseases with increased access to adequate sanitation facilities in schools. Ensuring ready access to safe drinking water, and hygienic toilets that offer privacy to users has great potential to beneficially impact children’s health. | 2012 | International Journal of Environmental Research and Public health | NA |
| 1. Social inequalities in educational facilities and structures | | | | | |
| Can preschool improve child health outcomes? A systematic review | D'Onise et al. | The aim of this study was to examine the evidence for child health effects of centre-based preschool intervention programs for healthy 4-year-olds, beyond the preschool years. The review found generally null effects of preschool interventions across a range of health outcomes, however there was some evidence for obesity reduction, greater social competence, improved mental health and crime prevention. We conclude that the great potential for early childhood interventions to improve population health across a range of health outcomes, as anticipated by policy makers worldwide, currently rests on a rather flimsy evidence base. Given the potential and the increasingly large public investment in these interventions, it is imperative that population health researchers, practitioners and policy makers worldwide collaborate to advance this research agenda. | 2010 | Social sciences Medecine | Early childhood education and care setting |
| Effectiveness of Early Educational Intervention | Barnett, WS | Early educational intervention has been proposed to partially offset the impacts of poverty and inadequate learning environments on child development and school success. A broad range of early educational interventions are found to produce meaningful, lasting effects on cognitive, social, and schooling outcomes. However, all interventions are not equally effective. Two major U.S. programs perform relatively poorly. Research provides some guidance regarding the features of highly effective programs, but much remains to be learned. New experimental studies of key program features would have a high payoff. | 2011 | Science | Early childhood education and care setting |
| Early Care, Education, and Child Development | Phillips et al. | Research aimed at identifying the short- and long-term impacts of ECEC experiences has a long history, the results of which now point to three key conclusions : i) although parents are the most important influence on children's development, ECE experiences have both short- and long-term impacts on a wide range of developmental outcomes that are best understood in interaction with family effects; b) the quality of adult-child interactions in ECE settings is the most potent source of variation in child outcomes, although the amount of exposure to these settings also plays a role, perhaps especially with regard to social-emotional development; iii) Some children, notably those growing up in poverty, appear to be more vulnerable to variation in the quality of ECE settings than do other children. | 2011 | Annual review of psychology | Early childhood education and care setting |
| Girls, mental health and academic achievement: a qualitative systematic review | Stentiford et al. | In this systematic review, we map the reported achievement-related factors affecting girls’ mental health emerging from the peer-reviewed qualitative literature. The themes of fears for the future, parent/family-related pressures, competitive school cultures, and gendered expectations of girls’ academic achievement emerged from the located texts. It was when pressures were “imbalanced” and felt in the extreme that mental ill-health/anxiety was more likely to be experienced. We go on to introduce the theoretical model of the “mental health/achievement see-saw” and argue for its use as a conceptual tool to engage with deep-rooted complexities around the relationship between gender, mental health and academic achievement. in schools that move beyond more surface-level attempts at mental health promotion and that seek to empower, de-pathologise and challenge entrenched structural inequalities. | 2021 | Educational review | Primary school / Secondary school |
| Reframing High-Quality Public Preschool as a Vehicle for Narrowing Child Health Disparities Based on Family Income | Martin et al. | Drawing on the bioecological paradigm of human development, we propose a model identifying specific mechanisms likely to promote equity in child health. These mechanisms reflect core characteristics of high-quality public preschool that may disproportionately benefit low-income children's health. This model serves as a working template for a program of future research. | 2021 | Academic Pediatrics | Early childhood education and care setting |
| Caste and control in schools: A systematic review of the pathways, rates and correlates of exclusion due to school discipline | Welsh et al. | This study provides a systematic review of the interdisciplinary literature on the relationship between school exclusion and students' short- and long-term educational and life outcomes. Although there are a handful of possible pathways, the most frequent pathway though which school discipline results in school exclusion are suspensions. The results of this systematic review indicate that school exclusion is not an efficacious response to student misbehavior given the short and long term correlates with negative student educational and life outcomes. There are several plausible mechanisms through which school exclusion may affect student outcomes but there is little empirical evidence on these mechanisms. | 2018 | Children and youth services review | Primary school / secondary school |
| What is the impact of structural and cultural factors and interventions within educational settings on promoting positive mental health and preventing poor mental health: a systematic review | Troy et al. | This review investigated which structural and cultural factors and interventions within educational settings promote positive MH and prevent poor MH in 4–18 years old. There was strong evidence for the impact of positive classroom management techniques, access to physical activity, and peer mentoring on student MH. Studies examining the impact of positive school culture, teacher training in MH and parent involvement in school MH activities also found predominantly positive results for student MH, albeit the evidence was of lower quality or from a low number of studies. Few studies explicitly examined the impact of interventions on MH inequalities; those that did indicated limited if any reduction to inequalities. A very small number of studies suggested that interventions targeting those at risk of poor MH due to socioeconomic factors could successfully improve wellbeing and reduce depression, anxiety and behavioural problems. Studies exploring the effect of management and leadership strategies within schools, policies, and aspects of the physical environment other than green space were scarce or absent in the literature. This review highlights the need to consider the ways in which educational settings are organised, the culture that is created and the physical space in order to improve the MH of CYP. | 2022 | BMC Public Health | Early childhood education and care setting / primary school / secondary school |
